# PARP inhibitors as potential therapeutic agents for various cancers: focus on niraparib and its first global approval for maintenance therapy of gynecologic cancers

**DOI:** 10.1186/s40661-017-0055-8

**Published:** 2017-11-29

**Authors:** Mekonnen Sisay, Dumessa Edessa

**Affiliations:** 1 Department of Pharmacology and Toxicology, School of Pharmacy, College of Health and Medical Sciences, Haramaya University, P.O.Box 235, Harar, Ethiopia; 2 Department of Clinical Pharmacy, School of Pharmacy, College of Health and Medical Sciences, Haramaya University, P.O. Box 235, Harar, Ethiopia

**Keywords:** PARP, PARP inhibitors, DNA repair, Cancer, Malignant tumors, Maintenance therapy, Niraparib, Mk-4827, Zejula, Companion diagnostic*

## Abstract

Poly (ADP-ribose) polymerases (PARPs) are an important family of nucleoproteins highly implicated in DNA damage repair. Among the PARP families, the most studied are PARP1, PARP2 and PARP 3. PARP1 is found to be the most abundant nuclear enzyme under the PARP series. These enzymes are primarily involved in base excision repair as one of the major single strand break (SSB) repair mechanisms. Being double stranded, DNA engages itself in reparation of a sub-lethal SSB with the aid of PARP. Moreover, by having a sister chromatid, DNA can also repair double strand breaks with either error-free homologous recombination or error-prone non-homologous end-joining. For effective homologous recombination repair, DNA requires functional heterozygous *breast cancer genes* (BRCA) which encode BRCA1/2. Currently, the development of PARP inhibitors has been one of the promising breakthroughs for cancer chemotherapy. In March 2017, the United States Food and Drug Administration (FDA) approved niraparib for maintenance therapy of recurrent gynecologic cancers (epithelial ovarian, primary peritoneal and fallopian tube carcinomas) which are sensitive to previous platinum based chemotherapy irrespective of *BRCA* mutation and homologous recombination deficiency status. It is the third drug in this class to receive FDA approval, following olaparib and rucaparib and is the first global approval for maintenance therapy of the aforementioned cancers. Niraparib preferentially blocks both PARP1 and PARP2 enzymes. The daily tolerated dose of niraparib is 300 mg, above which dose limiting grade 3 and 4 toxicities were observed. In combination with humanized antibody, pembrolizumab, it is also under investigation for those patients who have triple negative breast cancer. By and large, there are several clinical trials that are underway investigating clinical efficacy and safety, as well as other pharmacokinetic and pharmacodynamic profiles of this drug for various malignancies.

## Introduction

### General principles of DNA repair

**Fig. 1:**
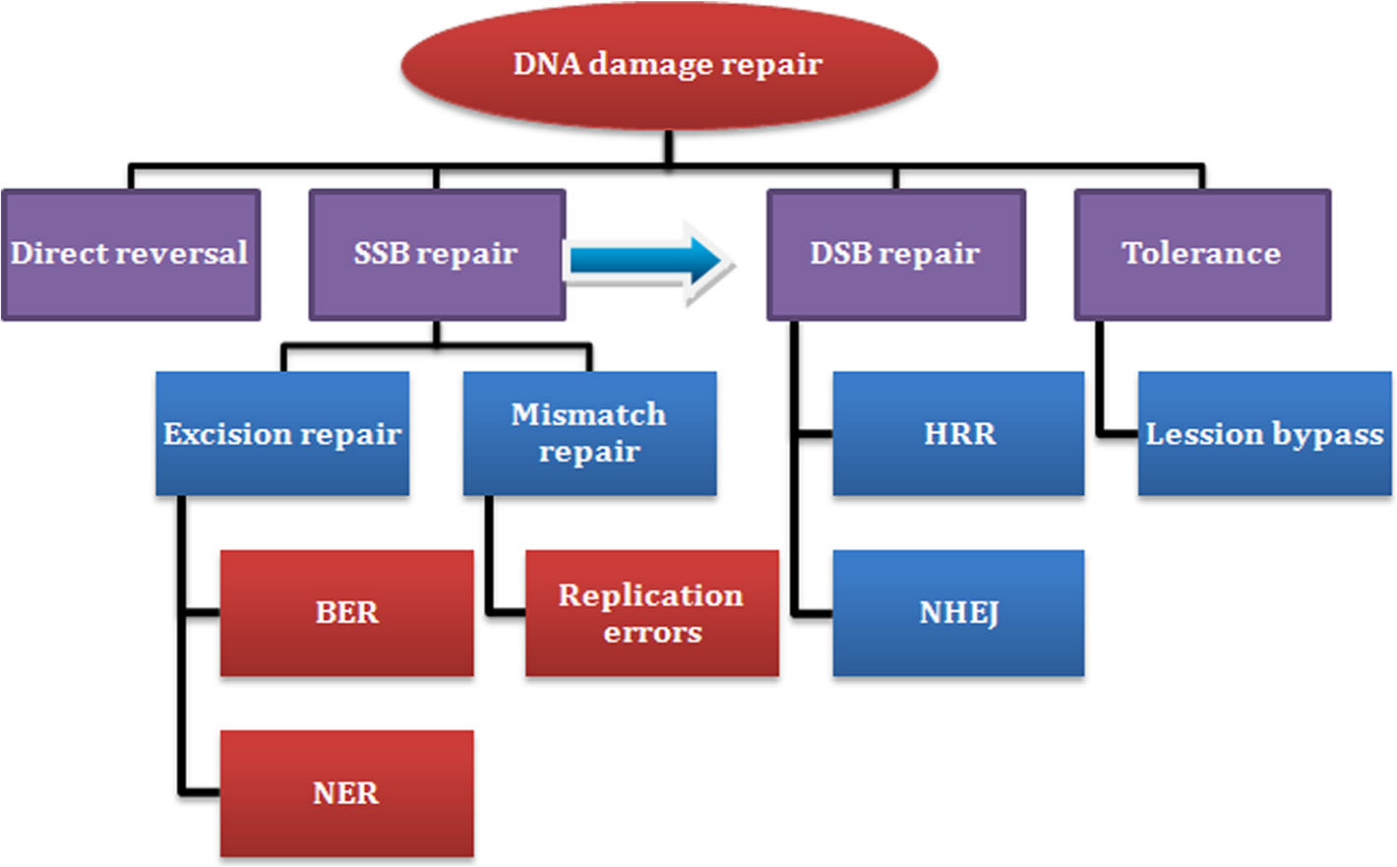
Illustrated diagram describing DNA repair pathways. (Note: BER, base excision repair; DSB, double strand break; HRR, homologous recombination repair; NER, nucleoside excision repair; NHEJ, non-homologous end-joining; SSB, single strand break)

The human genome is constantly under stress due to insults from both endogenous (free radicals or reactive oxygen species derived from metabolic processes) and exogenous (irradiation, chemicals, clinical drugs, and viruses, among others) sources. This results in routine DNA damage that may in turn lead to a serious genetic instability and cell death if it is left unrepaired. Being double stranded and having a sister chromatid, DNA repairs itself prior to cell division in any one of the following repair mechanisms (Fig. 1) [1–3].

Direct reversal is highly efficient, applicable when there is a single lesion, and is essentially error-free. The lesion may be tolerated or bypassed if it does not have a significant risk on the ongoing DNA replication and the genetic stability in general. Coming to the single strand break (SSB) repair, since the damage involves one strand of replicating DNA, it can be repaired by undergoing either excision of damaged site (base or nucleotide) or correcting the mismatch bases complementary to the anti-sense (template) strand [2, 4, 5].When a SSB is left unrepaired due to genetic and/or epigenetic factors, it will progress to double strand break (DSB) during DNA replication. By having homologous chromosomes, DNA has a back up to undergo DSB repair either by error-free homologous recombination (HR) or error-prone non-homologous end-joining (NHEJ). If the DSB is left unrepaired, it leads to a breakdown of the chromosome into smaller fragments, genomic instability, cell cycle arrest and apoptosis [1, 4, 6, 7]. The more faithful process of HR repair of DSBs involves localization of BRCA-1 and BRCA-2 proteins encoded from *breast cancer gene* to sites of DNA damage, resection of the DSB, and gap-filling DNA synthesis using the homologous sister chromatid as a template [8]. Before the DNA enters the repair process, cellular response depends upon the magnitude of the damage, resulting in induction of cell-cycle checkpoint pathways and DNA repair mechanisms. G2/M check point is a critical point where DNA must be repaired before the cell enters cell division/mitosis. If the damage is extensive and irreparable, induction of cell death occurs [3, 7].

### The role of poly (ADP-ribose) polymerases (PARPs) in DNA repair

**Fig. 2:**
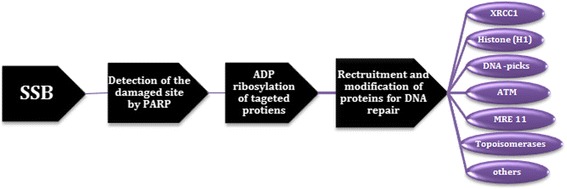
DNA repair processes with the aids of poly (ADP-ribose) polymerase. (Notes: XRCC1, X-ray cross complementing protein 1; ATM, ataxia telangiectasia-mutated kinase; MRE11, mitotic recombination 11. Others include: - Nijmegen breakage syndrome 1 (Nbs1), DNA ligase III, and DNA polymerase β)

PARPs are a member of nuclear protein enzymes highly implicated in DNA damage repair. During SSB, PARP detects the damaged site and undergoes post translational modification of targeted proteins by the process known as ADP-ribosylation. This process creates a conducive environment for recruiting several DNA repair proteins including topoisomerases, DNA ligase III, DNA polymerase β, and scaffolding proteins such as X-ray cross complementing protein 1 (XRCC1), among others. The ribosylation process also leads in relaxation of tightened chromatins and histones and results in unwinding of DNA to make it accessible for repair processes. What is more, PARP facilitates HR by recruiting factors such as ataxia telangiectasia-mutated kinase (ATM), mitotic recombination 11 (Mre11), and Nijmegen breakage syndrome 1 (Nbs1) to sites of DSBs (Fig. 2) [9–11]. When PARP activity is compromised, these SSBs cannot be repaired and progress to DSBs at DNA replication forks. In a normal cell, there is a cellular backup by which DSBs are repaired with the aid of HR, a mechanism different from base excision repair (BER) and hence, even in the absence of PARP activity and loss of BER, DNA repair can be effectively taken place by this pathway. However, cells can have a double-hit whereby both BER and HR are compromised. These cells rely on error-prone NHEJ for damage repair, which results in DNA instability and chromosomal aberrations, eventually resulting in apoptosis. The dual-insult of HR and BER defects results in “synthetic lethality” justifying the potent and lethal synergy between these two otherwise non-lethal event when they occur alone [1, 11]. Therefore, this review aims to address the role of common PARP inhibitors on cancer chemotherapy with special focus on niraparib and its first global approval for maintenance therapy of gynecologic cancers.

## Methods

A total of 945 articles were retrieved from various legitimate data bases and indexing services (Directory of open access journals, PubMed, PubMed Central, MEDLINE, Scopus and ProQuest), as well as other supplemental sources and search engines (CrosRef, WorldCat, and Google Scholar) with the aid of key terms: “PARP”, “PARP inhibitors”, “DNA repair”, “cancer”, “malignant tumors”, “Niraparib”, “MK-4827”, “Zejula”, “maintenance therapy” and “companion diagnostic*”. Boolean operators (AND, OR, NOT) were appropriately used for increasing the chance of obtaining relevant literature for this topic. Moreover, truncation was also applied to expand the literature searches and increase the number of related articles for inclusion. Following database searches and in-depth screening of each article by authors, majority of articles were removed from this study including duplicate articles from various databases and search engines; unrelated titles and abstracts; abstracts without full texts and full texts with insufficient information for data extraction. Finally, 68 references were included for the study from which, 22 articles were critically reviewed to summarize the current therapeutic profile of niraparib. Coming to the data extraction process, general background information concerning the role of PARP in DNA repair, cancer therapeutics and earlier inhibitors of this enzyme (s) were highlighted. Coming to the drug of interest, niraparib, data regarding the chemistry, pharmacology, primary outcomes of preclinical studies as well as completed clinical trials were extracted from respective individual studies. Furthermore, important data about ongoing clinical trials were also retrieved upon visiting https://clinicaltrials.gov/ web site. Data were collected from June to August, 2017.

## Review

### The role of PARP inhibitors in cancer chemotherapy

**Fig. 3:**
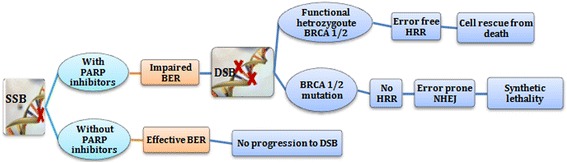
The influence of PARP inhibitors and BRCA mutation status in DNA repair and apoptosis of cancer cell. (Note: SSB, single strand break; BER, base excision repair; DSB, double strand break; HRR, homologous recombination repair; NHEJ, Non-homologous end-joining)

Ovarian cancer is the 5^th^ leading cause of cancer-related deaths in women in the United States. It is estimated that in 2017, more than 22,440 women will be diagnosed with ovarian cancer leading to more than 14,080 deaths. However, cancer chemotherapy has shown little improvement over time [12, 13]. So far, recurrent ovarian cancer has been dichotomized into two categories based on the sensitivity of platinum based therapy as ‘platinum sensitive’ and ‘platinum resistant’. This classification considers the number of months within which the patient can be freed from platinum based therapy from the last time of infusion to recorded recurrence. From molecular perspective, there is no clear cut demarcation to divide cancers based on sensitivity to platinum chemotherapy. With advancement in science and technology, targeted therapies like PARP inhibitors have led to a more holistic approach to the treatment of disease recurrence [14–16]. It is estimated that approximately 50% of high-grade serous ovarian cancer (HGSOC) show alterations in the *Fanconi anemia–BRCA* pathway [17]. Mutations in this pathway, including genes involved in HR repair such as RAD51C/D, and BRIP1 have been associated with homologous recombination deficiency (HRD) and hereditary ovarian cancer [18]. Epigenetic mechanisms can also contribute to the development of HRD. For example, silencing of BRCA1 in HGSOC has been shown to occur via epigenetic changes such as hypermethylation of BRCA1 promoter [17]. PARP inhibitors have been developed in the recurrence and maintenance treatment settings in epithelial ovarian cancer. As they inhibit SSB repair, inducing synthetic lethality in cells with underlying HRD as seen in BRCA1/2 mutant tumors (Fig. 3). Marked responses have been observed in ovarian cancers with BRCA1/2 mutation, even if up to 50% of HGSOC having HRD may also be better treated compared to cancers with HRD negative (HR proficient) genotypes [19, 20].

Among the newly diagnosed ovarian cancers, around 25% of them carry BRCA1/2 mutations from which majority (18%) are germline mutations whereas the remaining (7%) cancers are associated with somatic mutations [21]. In the absence of either germline or somatic mutations of BRCA1/2, HRD can occur in a variety of mechanisms as studied in several serious malignancies. The HR defects that occur due to aberration in genes other than BRCA exhibit closely resembling phenotypic characteristics secondary to PARP inhibitors and the condition is referred to as ‘BRCA-ness. This has been clearly demonstrated in either genetic mutations of ATM, RAD51C/D, check point kinases 2 (CHK2), phosphatase and tensin homolog (PTEN) or epigenetic silencing of BRCA1/2 promoter as having difficulty of effectively repairing DSBs by HR [22–25].

### Overview of common PARP inhibitors that got FDA approval

Beginning from the first efficacious in vitro study of PARP inhibitors, several agents have been studied in ovarian cancer [26, 27]. The best studied include olaparib, veliparib, talazoparib, rucaparib and niraparib (Table 1). Each PARP inhibitor possesses subtly different targets of PARP isoenzymes [28, 29]. PARP-1 is the most abundant and founding member of poly ADP-ribosylating proteins (a family of around 17 proteins) known as the ADP-ribosyltransferase diphtheria toxin-like proteins [16, 30]. In addition to the canonical targets of niraparib, PARP1 and PARP2, subsequent functional validation suggested that inhibition of deoxycytidine kinase by it could have detrimental effects when combined with nucleoside analogs used for the treatment of various diseases [31].

### Olaparib

The PARP inhibitor olaparib (Lynparza®) was the first to be approved in advanced ovarian cancer therapy for those with gBRCA1/2 mutations. Following phase I safety and efficacy studies, a multicenter phase II study demonstrated response to olaparib in patients with gBRCA1/2 mutations in recurrent ovarian cancer and breast cancer with at least 3 prior chemotherapy regimens. A subgroup analysis of patients with advanced ovarian cancer patients revealed an overall response rate (ORR) of 34% [32, 33]. These findings led to the fast track approval of olaparib capsules in the USA in December, 2014 as fourth line therapy. The approval of olaparib for advanced ovarian cancer patients with BRCA 1/2 mutation and who are intensively pretreated with chemotherapy becomes a major therapeutic breakthrough for this lethal and difficult to treat disease. Even if it is the first agent in its class to get fast track approval by FDA, rucaparib and niraparib received recent approval in slightly different settings. Several PARP inhibitors are also under clinical development either alone or in combination with other treatment modalities including radiation therapies, cytotoxic agents and antiangiogenic agents [6] (Table 1). On August 17, 2017, FDA granted regular approval to olaparib tablets (Lynparza, AstraZeneca) for the maintenance treatment settings of adult patients with recurrent gynecologic cancers, who are in a complete or partial response to platinum-based chemotherapy [34]. The recent approval of olaparib in the maintenance setting was based on two randomized, placebo-controlled, double-blind, multicenter trials in patients with recurrent gynecologic cancers who were in response to platinum-based therapy.

**Table 1:** Overview of common FDA approved and investigational PARP inhibitors and their treatment profile

Name of the drug	Approval by FDA	Clinical conditions for which the drug are approved or under investigation	Route	Targeted PARP enzyme(s) (Affinity)	IC_50_	Line of previous chemotherapy	References
Veliparib (ABT-888)	Under investigation (its efficacy and safety have not been established yet)	FDA grants orphan drug designation for advanced squamous non-small cell lung cancer (Phase III)	PO	PARP 1and PARP2	5.2 nM/2.9 nM (PARP1/2)	_____	[63]
Fluzoparib (SHR3162)	Under investigation (phase I) in combination with apatinib	Recurrent ovarian cancer	PO	PARP 1 and PARP 2	______	Two lines of platinum-based therapy (gynecologic cancers) and only one line of standard chemotherapy (TNBC)	[64]
		TNBC					
Talazoparib (BMN 673)	Investigational drug	Under development for advanced breast cancer patients with gBRCA mutations	PO	PARP 1/2>>>>PARP3	1.2 nM/0.9 nM (PARP1/2)	_____	[65]
Olaparib (Lynparza®)	December 2014 (First FDA approval)	Patients with germline BRCA1/2-mutated advanced recurrent ovarian cancer	PO	PARP 1 > PARP2>>PARP3	5 nM/1 nM(PARP1/2)	≥ 3 prior lines of chemotherapy	[9, 32, 33].
	Approved again on Aug 17, 2017	For the maintenance treatment of adult patients with recurrent gynecologic cancers	PO	______	______	≥ 2 lines of therapy	[34]
Rucaparib (Rubraca®)	December 2016 (Second approval)	Treatment of ovarian cancer patients with somatic and/or germline BRCA mutations	PO	PARP 1>>>>>PARP2/3	1.4 nM (PARP1)	One line earlier than olaparib (patients who have received ≥2 prior lines of chemotherapy)	[37, 38, 60]
	Sought FDA approval for second time on October 10, 2017	For maintenance treatment settings	PO	________	______	≥ 3 lines of therapy	[40]
Niraparib (Zejula™)	March 2017 (Third Approval)	Maintenance therapy of adult patients with recurrent gynecologic cancers irrespective of the status of BRCA mutations and/or HRD status	PO	PARP1 and PARP2	3.2 nM/4 nM (PARP1/2)	- CR or PR to previous (at least two) platinum-based chemotherapy.	[42, 56]

Abbreviations: *IC* intracellular concentrations, *BRCA* breast cancer genem, *PO* per oral, *PARP* Poly(ADP-ribose) polymerase, *CR* complete response, *PR* partial response, *FDA* Food and Drug Administration, *HRD* homologous recombination deficiency, *TNBC* triple negative breast cancer

Study 19 (NCT00753545), a phase II trial engaged 265 patients with platinum sensitive HGSOC regardless of BRCA status (1:1) to receive olaparib capsules 400 mg orally twice daily or placebo. Study 19 demonstrated a statistically significant improvement in investigator-assessed progression-free survival (PFS) in patients treated with olaparib compared to placebo (Hazard Ratio (HR') = 0.35; 95% CI: 0.25, 0.49; *p* < 0.0001). The estimated median PFS was 8.4 months and 4.8 months in the olaparib and placebo arms, respectively. Clinical outcomes between placebo- and olaparib-treated patients with somatic BRCA1/2 mutations were similar to those with germline BRCA1/2 mutations, indicating that patients with somatic BRCA1/2 mutations benefit from treatment with olaparib in maintenance setting [35]. SOLO-2/ENGOT-Ov21 (NCT01874353), a phase III clinical trial, randomized 295 patients with recurrent germline BRCA-mutated gynecologic cancers (2:1) to receive olaparib tablets 300 mg orally twice daily or placebo. SOLO-2 demonstrated a statistically significant improvement in investigator-assessed PFS in patients randomized to olaparib compared with those who received placebo (HR' = 0.30; 95% CI: 0.22, 0.41; *p* < 0.0001). The estimated median PFS was 19.1 and 5.5 months in the olaparib and placebo arms, respectively [36].

### Rucaparib

Coming to rucaparib (Rubraca®), it is a potent inhibitor of PARP1, PARP2 and PARP 3 with the greatest affinity towards PARP1. Both phase I and II clinical trials demonstrated that it has a promising efficacy in ovarian cancers with both BRCA mutation (including germline and somatic subtypes) and tumors with HRD positive. In December, 2016, US FDA approved rucaparib for the treatment of advanced ovarian cancer patients with general BRCA1/2 mutation and who took at least 2 lines of previous platinum based therapy. The accelerated approval was based upon ORR (54%) [37]. In ARIEL2 part 1, patients with recurrent, platinum-sensitive, high-grade ovarian carcinomas were classified into one of three predefined HRD subgroups on the basis of tumor mutational analysis: *BRCA* mutant (deleterious germline or somatic), *BRCA* wild-type and loss of heterozygosity (LOH) high (LOH high group), or *BRCA* wild-type and LOH low (LOH low group). Median PFS after rucaparib treatment was 12·8 months (95% CI 9·0–14·7) in the *BRCA* mutant subgroup, 5·7 months (5·3–7·6) in the LOH high subgroup, and 5·2 months (3·6–5·5) in the LOH low subgroup. PFS was significantly longer in the *BRCA* mutant (HR' = 0·27, 95% CI 0·16–0·44, *p* < 0·0001) and LOH high (0·62, 0·42–0·90, *p* = 0·011) subgroups compared with the LOH low subgroup. Part 2 of the ARIEL2 trial is ongoing, and it will prospectively evaluate rucaparib responsiveness in patient sub-groups defined by LOH scores [38].

ARIEL3 (NCT01968213), a randomised, double-blind, placebo-controlled, phase 3 trial, demonstrated improved PFS by investigator review for rucaparib compared with placebo in all three primary efficacy analyses: BRCA mutation (16.6 months vs. 5.4 months; HR': 0.23, *P* < 0.001); HRD-positive (13.6 months vs. 5.4 months; HR': 0.32, *P* < 0.001); overall intent-to-treat populations (10.8 months vs. 5.4 months; HR': 0.36, P < 0.001) [39]. On October 10, 2017, FDA approval was sought for maintenance therapy of rucaparib in ovarian cancer following promising findings from ARIEL3 clinical trial (Table 1) [40].

### Niraparib

Among the PARP inhibitor series, niraparib (Zejula) is the third drug in this class, to receive FDA approval for cancer chemotherapy. However, the previous PARP inhibitors, olaparib and rucaparib have been approved for simple treatment rather than maintenance for those patients who are responsive to previous chemotherapy. Niraparib has become the first drug that got global approval for maintenance therapy of patients with recurrent gynecologic cancers regardless of their BRCA mutation and HRD status [41, 42] (Table 1). Hereafter, this review  focuses on summarizing the chemistry, pharmacology, preclinical studies, completed and ongoing clinical trials as well as toxicological concerns of niraparib.

### Chemistry, pharmacology and preclinical data of niraparib

**Fig. 4:**
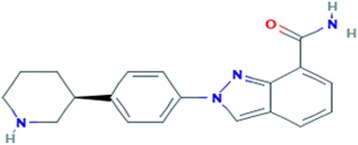
Chemical structure of niraparib

Niraparib (Zejula, MK-4827; (2-[4-[(3S)-piperidin-3-yl], phenyl]indazole-7-carboxamide) is a potent and selective inhibitor of PARP-1 and PARP-2 enzymes. Its molecular formula is C_19_H_20_N_4_O and has a molar mass of 320.396 g/mol (Fig. 4) [43].

Niraparib is administered orally on once daily basis and can be taken without consideration to meals since food does not significantly affect the absorption and/or the metabolism of niraparib [44]. It is readily absorbed from the oral route and its bioavailability is approximately 73% in humans as per the phase III clinical trials revealed. There is no significant difference in pharmacokinetic parameters between the feeding and fasting states. For example, the mean ratios of maximum plasma concentration (C_max_) and area under the curve (AUC_0-∞_) in the fed to fasted state were 0.83 and 1.08, respectively. In both cases, niraparib possesses long terminal half-life (t_1/2_) of greater than 2 days (57 and 59 h for feeding and fasting states, respectively). This is consistent with once daily dosing of niraparib for cancer chemotherapy [45]. Coming to the metabolic profile of niraparib, it had been shown that niraparib is moderately metabolized in humans primarily via hydrolytic (phase I) and conjugative (Phase II) pathways in the liver. The hepatic phase I metabolism is via carboxylesterase-catalyzed amide hydrolysis, leading to the formation of inactive metabolite (carboxylic acid derivative) which in turn undergoes a phase II conjugation reaction called glucuronidation for ease of biliary and renal excretion [46]. Unlike rucaparib and olaparib, studies indicated that cytochrome P-450 enzymes (CYP) including CYP 1A2 play a negligible role in the metabolism of niraparib in humans [46–48]. Moreover, from the total administered dose, 31.6% and 40.0% are recovered in feces and urine, respectively, whereas 29.9% of the dose is excreted unchanged in the urine and feces [46].

In preclinical trial of rodent’s model, it was also indicated that almost similar concentration-time profile of niraparib was obtained from both brain and plasma samples, and the mean brain-to-plasma concentration ratios following a single oral dose ranged from 0.85–0.99 of the brain T_max_ (Table 2) [49]. Moreover, different preclinical and clinical studies indicated that niraparib induces chemo- and radio-sentiziation and hence facilitates cell death in cancer cells. Combinational therapy of niraparib with topoisomerase inhibitors such as irinotecan chemosensitize cancer cells as observed both in in-vivo and in vitro studies. In several breast and lung cancer models, niraparib enhances the therapeutic effects of radiation therapy independent of the tumor suppressor P-53 function (Table 3). Having impaired the BER function by niraparib, exposure of radiation converts the sub-lethal SSBs into lethal DSBs leading to synthetic lethality of cancer cells [50–52].

**Table 2:** Overview of the pharmacokinetic profile of niraparib in preclinical and clinical studies

Description of study population	Methods	Results	References
Patients with ovarian cancer	Two-way crossover design ((feeding versus fasting) - each subject received 2 separate 300-mg doses of niraparib, 1 each in a fasting and a fed state - investigating pharmacokinetic parameters based on feeding state	- The mean ratios of C_max_ and AUC_0-inf_ in the fed (test) versus fasted state (reference) were 0.83 and 1.08, respectively - The mean t_1/2_ of feeding and fasting states are 57 and 59 h, respectively - Median T_max_ in the feeding condition is almost 2 times to that of fasting state	[45]
Rodents with BRCA2-mutant (Capan-1) and MDA-MB-436 (BRCA-1 mutant) human pancreatic cancer xenograft model	Randomized cohorts of Balb/c nude mice bearing either subcutaneous Capan-1 tumors, or intracranial Capan-1-luc tumors - Dosing of niraparib (15, 30, or 45 mg/kg QD) - Up to 50 days - Investigating the brain and plasma levels of niraparib	- Similar Concentration-time profiles of niraparib in the brain and plasma - Mean brain-to-plasma concentration ratios for niraparib following a single oral dose to rats were 0.85–0.99 of the brain Tmax - Brain Ctrough levels (24 h) were 2–4 times greater than observed in plasma, indicating niraparib is able to penetrate the brain in rodents - Have therapeutic benefit in an IC BRCA-mutant human xenograft model	[49]

Abbreviations: *QD* every day, *BRCA* breast cancer, *IC* intracranial

The activity of niraparib in sporadic prostate cancer provides a strong clinical evidence for developing PARP inhibitor based therapies for metastatic castration resistant prostate cancer (CRPC). Erythroblast transformation-specific (ETS) gene rearrangement and loss of PTEN are among the common genetic alterations in prostate cancer and have been linked to increased sensitivity to PARP inhibitors in preclinical models [4]. Moreover, compared to other PARP inhibitors, niraparib was found to be effective as a monotherapeutic agent in several cell lines tested in pediatric diffuse intrinsic pontine glioma and pediatric high-grade astrocytoma [53].

**Table 3:** Preclinical studies of niraparib on different cancer models

Study characteristics	Methods	Primary outcomes observed	References
Panel of 25 TNBC PDX models in mice	Gapped sequential design (cyclophosphamide followed by niraparib after 14 days) ✓ investigating the antitumor efficacy of niraparib alone or in combination with alkylating agent, cyclophosphamide (standard chemotherapy of TNBC)	- Cyclophosphamide showed partial to complete tumor regression - For niraparib, significant antitumor response occurs with BRCA mutations or a high HRD score - Potentiation with inhibition of tumor relapse after discontinuing cyclophosphamide (in niraparib sensitive tumor sub types) - In niraparib responder cells, superior efficacy compared to sequential therapy of cyclophosphamide alone	[66]
Panel of 17 BBC PDXmodels in mice	- Experimental design in which groups were treated with niraparib (50 mg/kg/day) and vehicle control separately - 13 of BBC were TNBC cells - Treatment continued for 28 days - Tumor volume and body weight measurements	- No sign of body weight reduction relative to the vehicle control - Niraparib exhibited robust efficacy in five of the 17 models. All five responsive models were TNBC - Niraparib is generally effective in subset of TNBC patients	[67]
Four neuroblastoma cell lines (in vitro) and a murine xenograft model of metastatic neuroblastoma (in vivo)	- Clonogenic survival assays - ELISA (PARP assay) ○ Poly ADP - Immunohistochemistry ✓ Measurement of cleaved caspase-3, γ-H2AX, and Ki67	- Reduced clonogenicity - Additive effects with radiation - Significantly prolonged survival in combined modalities - ↑cleaved caspase-3 and γ-H2AX	[68]
Tumor cell lines derived from lung, breast, and prostate cancers (MDA-MB-231, LnCaP, MDA-MB-436, CCD-16, and MCF-10A cells) plus normal cell lines	- Clonogenic survival analyses	- μM conc of niraparib radiosensitized tumor cell lines independently of their p53 status but not cell lines derived from normal tissues. - It also sensitized tumor cells to H_2_O_2_	[50]

Abbreviations: *TNBC* triple negative breast cancer, *ADP* adenosine diphosphate, *ELISA* enzyme linked immunosorbet assay, *HRD* homologous recombination deficiency, *PDX* patient derived xenograft, *BBC* basal breast cancer, *↑* increased

### Evidences from clinical trials of niraparib

Niraparib is approved for the maintenance therapy of adult patients with recurrent gynecologic cancers that are in a complete or partial response to previous platinum-based chemotherapy. In Europe, it is under review by European Medicine Agency for maintenance therapy of recurrent ovarian cancer patients who are sensitive to earlier platinum chemotherapy [42].

In a phase 1 dose-escalation study, 100 patients with advanced solid tumors were enrolled in two parts. In part A, cohorts of three to six patients, enriched for *BRCA1* and *BRCA2* mutation carriers, received niraparib daily at ten escalating doses from 30 mg to 400 mg in a 21-day cycle to establish the maximum tolerated dose. In part B, further investigation was conducted to determine the maximum tolerated dose in patients with sporadic platinum-resistant high-grade serous ovarian cancer and sporadic prostate cancer. Considering various side effects associated with dose escalation, 300 mg/day was established as the maximum tolerated dose (Table 4). Niraparib was found to inhibit tumor growth in models with loss of BRCA activity and loss of function mutation of tumor suppressor PTEN proteins. Sandhu et al. administered niraparib to a small cohort of patients enriched for BRCA-deficient and sporadic cancers associated with defects in HR repair. Thirty-nine patients were treated, 11 of whom had gBRCA1/2 mutations. Eight of the BRCA1/2 mutation carriers with ovarian cancer had a partial response. What is more, antitumor activity was also found in sporadic HGSOC [54].

**Table 4:** Clinical trials of niraparib for cancer patient with different histological subtypes

Description of Study participants	Phases	Methods	Observed outcomes (primary and/or secondary)	References
Hundred patients with advanced solid tumors in three sites ((dose escalation study)	phase I	Two cohort studies with single arm in each Part A: 60 patients • enriched for BRCA1 and BRCA2 mutation carriers • received niraparib daily at ten escalating doses from 30 mg to 400 mg in a 21-day cycle to establish the maximum tolerated dose Part B: 40 patients • sporadic platinum-resistant HGSOC and sporadic prostate cancer • investigating the maximum tolerated dose	- maximum tolerated dose is 300 mg/day dose liming toxic effects (Initial cycle) - Grade 3 fatigue (30 mg/day) and pnemonitis (60 mg/day) were observed during first cycle - Grade 4 thrombocytopenia (400 mg/day) - Other common treatment related grade 1 and 2 side effects - Inhibition of PARP exceeds 50% at dose greater than 80 mg/day (80 mg > ED50) - Antitumor effect was observed beyond 60 mg/day	[54]
Patients with sporadic CRPC	Phase I	randomized clinical trial with two treatment arms (21 patients) ‑ Arm 1: niraparib 290–300 mg/day ‑ Arm 2: placebo	- Stabilization of CRPC - in 43% of patients with a median duration of response of 254 days - 30% of patients had a decrease of circulating tumor cells - No correlation between ERG rearrangements/loss of PTEN expression and treatment response.	[55]
Patients with recurrent OC (553 patients)	phase III	Randomized double blind clinical trial with two category ad two arms per category - Arm 1: Niraparib 300 mg once daily • 138 patients - Arm 2: placebo • 65 patients ➢ Both arm 1 and 2 patients are with gBRCA mutant tumors - Arm 3: Niraparib 300 mg once daily • 234 patients - Arm 4: Placebo • 116 patients ➢ Both arm 3 and 4 patients are with non-gBRCA mutant (wild type) tumors irrespective of HR status	The primary outcomes were PFS In gBRCA cohort • (21 months vs. 5.5 months for treatment to placebo) For non-gBRCA cohort with HRD positivity • The PFS was found to be 12.9 and 3.8 months for niraparib and placebo arms, respectively. Overall PFS in non-gBRCa cohor • 9.3 months vs 3.9 months	[56]
181 patients with recurrent OC, no prior use PARP inhibitors and at least 2 previous platinum therapy	Phase III	Randomized double blind clinical trial (two cohorts based on gBRCA status)	Platinum resistance rates were 42%, 53% and 49% for gBRCA, non-gBRCA and pooled cohorts, respectively	[57]

Abbreviations: *HGSOC* high grade serious ovarian cancer, *PFS* progression free survival, *OC* ovarian cancer, *CRPC* castration resistant prostate cancer

While only a minority of prostate cancer patients carries germline mutations, many sporadic CRPCs harbor epigenetic and genetic disruption of genes that are crucial for the HR pathway including *BRCA1, BRCA2, FANC, ATM, CHEK1/2, MRE11A* and *RAD5*1. Some of these aberrations have been associated with responsiveness to PARP inhibitors and platinum, justifying a synthetic lethality between platinum or PARP inhibitors and these sporadic DNA repair gene defects [55] (Table 4).

In the multinational, randomized, double blind, phase III clinical trial (ENGOT-OV16/NOVA trial), adult patients were dichotomized in two cohorts, each containing two arms, based on the status of *gBRCA* mutation (gBRCA cohort and non-gBRCA cohort). The categorization was based on BRCAnalysis CDx_BRCA_ testing (Myriad Genetics, Salt Lake City, USA). Patients were randomly assigned in 2:1 ratio to receive 300 mg niraparib or placebo in each cohort. In this study, the primary end point (outcome) measured was the PFS. To further fine tune the efficacy of niraparib on different ovarian cancer histological subtypes, the non gBRCA cohort was further classified in to HRD positive (HR deficient) and HRD negative (HR proficient) based on myChoice HRD™ test (Myriad Genetics). In this trial, the total number of patients enrolled was 553 and from those 203 were assigned to gBRCA cohort (138 to treatment arm (niraparib) and 65 to placebo) while the remaining 350 patients were assigned to non gBRCA cohort (234:116 for niraparib to placebo group). In gBRCA cohort, patient in the niraparib arm had significantly longer median PFS period (21 months) compared to placebo group (5.5 months) [HR', 0.27; 95% CI: 0.17–0.4. Coming to the non gBRCA cohort with HRD positive patients, the median PFS was found to be 12.9 months and 3.8 months for niraparib and placebo groups, respectively [HR', 0.38; (95% CI: 0.24–0.59]. The overall PFS in non-gBRCA cohort regardless of HRD status was 9.3 months vs 3.9 months [HR', 0.45; 95% CI: 0.34–0.61]. Generally, from this clinical trial, we can conclude that niraparib can be given to recurrent ovarian cancer patients regardless of gBRCA and HRD status (Table 4) [56].

The successful phase 3 niraparib ENGOT-OV16/NOVA trial also included a substantial number of patients with platinum resistant ovarian cancer. In this study, 181 patients were assigned to placebo (65 gBRCAmuts and 116 non-gBRCAmuts). The prevalence of platinum resistance estimated for the gBRCAmut, non-gBRCAmut, and pooled cohorts were 42%, 53%, and 49%, respectively (Table 4). Approximately half of the patients in the NOVA study, where niraparib treatment met its primary endpoint by prolonging PFS following a response to platinum, had developed resistance at last line of chemotherapy (Table 4) [57].

### Toxicological concerns of niraparib

In phase I dose escalation trial, common treatment-related toxic effects were anemia (48%), nausea (42%), fatigue (42%), thrombocytopenia (35%), anorexia (26%), neutropenia (24%), constipation (23%), and vomiting (20%), and were predominantly grade 1 or 2. The most common grade 3 or 4 adverse events that were reported in the niraparib group were thrombocytopenia (33.8%), anemia (25.3%), and neutropenia (19.6%), which were managed with dose modifications [54]. Niraparib is also associated with serious risks, such as hypertension, hypertensive crisis, myelodysplastic syndrome, acute myeloid leukemia, and bone marrow suppression. Women who are pregnant or breastfeeding should not take niraparib because it may cause harm to a developing conceptus or a newborn baby [41, 56].

### Companion diagnostic tests

Companion diagnostic tests are very critical to identify cancer patients who are best treated by PARP inhibitors. Myriad’s BRCA analysis CDx™ is the only FDA-approved test to determine olaparib treatment eligibility. Rucaparib’s companion diagnostic test (FoundationFocus™CDx_BRCA_ test that detects germline and somatic BRCA1/2 mut) is the first FDA-approved next-generation sequencing (NGS)-based test designed to identify patients likely to respond to rucaparib [58–60]. Coming to niraparib, the eligibility is determined with myChoice HRD™ test (Myriad Genetics). While BRCA analysis CDx™, as the name explains, evaluates only BRCA, myChoiceHRD™, developed by the same company, evaluates LOH beyond BRCA and can be considered an enhancement of BRCA analysis CDx™. It is an NGS-based assay that assesses BRCA1/2 sequences, and genomic scarring (HRD score), composed by LOH, telomeric allelic balance and large-scale transitions [61].

### Ongoing clinical trials

There are several clinical trials that are underway for invesigating safety, tolerability, efficacy, and other pharmacokinetic and pharmacodynamic profiles of niraparib in different treatment modalities (single and/or combination therapies) and cancers of diverse histological origin. Several phase I clinical trials are underway investigating the maximum tolerated dose of niraparib when used in combiation with different treatment modalitites: with enzalutamide in CRPC, with everolimus in ovarian cancer, as well as with temozolomide and irinotecan in case of ewing sarcoma, among others. In phase II clinical trials, the safety and efficacy of niraparib alone is on the way to be investigated. Coming to the phase III clinical trials, the primary outcome measures of niraparib alone and in comparison with physician’s choice have been under evaluation for maintenance therapy of advanced ovarian cancer patients following a response to front line platinum based therapy and human epidermal growth factor receptor 2 negative (HER2-), gBRCA mut-positive breast cancer patients, respectively(Table 5) [62].

**Table 5:** Ongoing clinical trials of niraparib alone or in combination with other agents for treatment of various malignancies [62]

ClinicalTrials.gov Identifier	Title	Conditions under study	Phase	Interventions (Experimental arms)	Primary outcome measures of niraparib	Recruitment status
NCT03209401	Niraparib plus carboplatin in patients with HRD advanced solid tumor malignancies	Solid malignancies in adult patients with evidence of HRD	Phase 1	Niraparib Carboplatin	The dose of niraparib required to combine with carboplatin	Not yet recruiting
NCT03076203	Phase IB Trial of Radium-223 and niraparib in patients with CRPC (RAPARP)	Prostate carcinoma metastatic to the bone Stage IV prostate adenocarcinoma Hormone-refractory prostate cancer	Phase I	Niraparib Radium R_a_ 223 Dichloride	To determine MTD to combine with radiation	Not yet recruiting
NCT02500901	Enzalutamide and niraparib in the treatment of CRPC	Metastatic prostate pancer	Phase I	Enzalutamide Niraparib	MTD	Active, but not recruiting
NCT03154281	Evaluation of the safety and tolerability of niraparib with everolimus in ovarian and breast cancer	Breast cancer Ovarian cancer	Phase I	Niraparib Everolimus	MTD	Not yet recruiting
NCT02044120	ESP1/SARC025 global collaboration: a phase I study of a combination of the PARP inhibitor, niraparib and temozolomide or irinotecan in patients with previously treated, incurable Ewing sarcoma	Ewing sarcoma	Phase I	Niraparib Temozolomide Irinotecan	DLT and MTD	Recruiting
NCT02924766	A safety and pharmacokinetics study of niraparib plus an androgen receptor-targeted therapy in men with metastatic CRPC (BEDIVERE)	Prostatic neoplasms	Phase I	Niraparib Apalutamide Abiraterone Acetate Prednison	Determine Recommended Phase 2 dose	Recruiting
NCT03207347	A Trial of niraparib in BAP1 and Other DNA DSB repair deficient neoplasms (UF-STO-ETI-001)	Mesothelioma Uveal melanoma Renal cell carcinoma Cholangiocarcinoma	Phase II	Niraparib	ORR	Not yet recruiting
NCT02657889	Study of niraparib in combination with pembrolizumab (MK-3475) in patients with TNBC or Ovarian Cancer (TOPACIO)	TNBC Ovarian cancer Stage IV breast cancer Fallopian tube cancer Peritoneal cancer	Phase I/II	Niraparib Pembrolizumab	Evaluate DLT	Recruiting
NCT02354131	Niraparib versus niraparib-bevacizumab combination in women with platinum-sensitive epithelial ovarian cancer (AVANOVA)	Ovarian cancer	Phase I/II	Niraparib Bevacizumab	PFS	Recruiting
NCT02854436	An efficacy and safety study of niraparib in men with metastatic CRPC and DNA-Repair anomalies (Galahad)	Prostatic neoplasms	Phase II	Niraparib	ORR	Suspended
NCT02354586	A study of niraparib in patients with ovarian cancer who have received three or four previous chemotherapy regimens (QUADRA)	Ovarian cancer	Phase II	Niraparib	Evaluation of antitumor activity	Recruiting
NCT01905592	A phase III trial of niraparib versus physician’s choice in HER2-, germline BRCA mutation-positive breast cancer patients (BRAVO)	Breast cancer HER 2-breast cancer BRCA1/2 gene mutation	Phase III	Niraparib Physician’s choice	PFS	Active, but not recruiting
NCT02655016	A study of niraparib maintenance treatment in patients with advanced ovarian cancer following response on front-line platinum-based chemotherapy	Ovarian cancer	Phase III	Niraparib	PFS	Recruiting

Abbreviations: *ORR* Objective response rate, *DLT* dose limiting toxicity, *MTD* maximum tolerated dose, *HRD* homologous recombination deficiency, *TNBC* triple negative breast cancer, *CRPC* castaration resistant prostate cancer, *HER2* human epethilial growth factor receptor 2 negative

## Conclusion and future prospects

The role of PARP family enzymes in DNA repair and cancer therapeutics was well emphasized in this review article. The development of PARP inhibitors has become one of the promising breakthroughs and hot spots in the area of experimental oncology. As documented in various histological subtypes of cancer, there are several germline and/or somatic mutations, as well as epigenetic alterations compromising effective reparation of DSBs by HR repair. This will create medically important and selective situation whereby cancer cells will be subjected to dual insult of PARP inhibitors and mutations of HR genes including BRCA1/2. Normal cells are less likely to be affected by PARP inhibitors since they have functional HR for DSB. Based on this evidence, scientists are striving to discover PARP inhibitors which have superior safety and efficacy profiles than the existing medications for cancer chemotherapy. Even if niraparib is the third drug to get FDA approval from its class, it is the first one to receive global approval for maintenance therapy of patients with recurrent gynecologic cancers regardless of BRCA and HRD status. Maintenance therapy is an important part of cancer chemotherapy for patients who have responded positively to a primary treatment. Niraparib offers patients a new treatment option that may help delay the future growth of these cancers, regardless of whether they have a specific genetic mutation. Niraparib has also several important pharmacokinetic features including negligible interaction with food; once daily dosing regimen; less likely to interact with other coadministered drugs since it is primarily metabolized by hydrolytic and conjugative pathways, and lower dosage requirement than previously approved PARP inhibitors (olaparib and rucaparib). It is also a potent inhibitor of PARP 1 and PARP 2 enzymes. Evidence from randomized phase III clinical trials indicated that niraparib can be given to any ovarian cancer patients who are responsive to previous therapy. Additional feature here is that this drug can also be given to patients irrespective of HRD status: HRD negative (HR proficient) and HRD positive (HR deficient) cells as statistically significant median PFS was observed in niraparib arms of both cohorts compared to placebo. Generally, niraparib is under investigation either alone or in combination with other treatment modalities for several cancer types. Among them, niraparib alone is under study in phase III clinical trial for maintenance treatment of patients with advanced ovarian cancer following a response on front line platinum based therapy. In combination with pembrolizumab, it is at the transition of phase I/II trials investigating the dose limiting toxicity in triple negative breast cancer patients. Moreover, the efficacy (PFS) of niraparib in comparison with physicians’ choice is also under consideration in phase III (BRAVO) trial for HER2- breast cancer patients.

## Abbreviations

BER: Base Excision Repair
BRCA: Breast Cancer gene
CHK2: Check point Kinase 2
CRPC: Castration Resistant Prostate Cancer
DSB: Double Strand Break
ETS: Erythroblast Transformation-Specific gene
FDA: Food and Drug Administration
gBRCA: Germline mutation of Breast Cancer gene
HER2-: Human Epidermal growth factor Receptor 2 negative
HGSOC: High Grade Serious Ovarian Cancer
HR: Homologous Recombination
HR': Hazard Ratio
HRD: Homologous Recombination Deficiency
LOH: Loss of Heterozygosity
NHEJ: Non Homologous End Joining
PARP: Poly (ADP-Ribose) Ploymerase
PFS: Progression Free Survival
PTEN: Phosphatase and Tensin homolog
SSB: Single Strand Break
TNBC: Triple Negative Breast Cancer
